# Oribatid mites like lichens—feeding preferences among three *Cladonia* species with different chemistry

**DOI:** 10.1038/s41598-026-54891-5

**Published:** 2026-05-31

**Authors:** Věra Vtípilová, Tobias Pfingstl, Heda Ghlimová, Jan Mourek, Jana Steinová, Philipp Resl

**Affiliations:** 1 Department of Botany, Faculty of Science, Charles University, Benátská 2, 12801 Prague, Czech Republic; 2 Institute for Environmental Studies, Faculty of Science, Charles University, Benátská 2, 12801 Prague, Czech Republic; 3 Institute of Biology, University of Graz, Universitätsplatz 2, 8010 Graz, Austria; 4 Mycological Department, National Museum, Cirkusová 1740, 19300 Prague, Czech Republic; 5 Department of Biology Education, Faculty of Science, Charles University, Viničná 7, 12843 Prague, Czech Republic; 6 Institute of Biology, University of Graz, Holteigasse 6, 8010 Graz, Austria

**Keywords:** Acari, Fumarprotocetraric acid, Rhodocladonic acid, Feeding tests, *Carabodes*, Ecology, Ecology, Zoology

## Abstract

Lichens in general and members of the genus *Cladonia* in particular are known for their repertoire of chemical substances. These substances have been attributed various roles, including a role in the defence against herbivores. Unsurprisingly, *Cladonia* species share habitats with many small invertebrates, like mites. Mites have been suggested to regularly feed on lichens, with potential detrimental effects for the lichen. Here we tested the feeding preferences of two oribatid mite species (*Carabodes areolatus* and *C. marginatus*) for three members of the lichen genus *Cladonia*. *Cladonia coniocraea*, *C*. *norvegica* and *C*. *rubrotincta* have similar morphology, ecology, and they regularly share their habitats with the selected mites, but produce different chemical compounds (barbatic acid in *Cladonia norvegica*, barbatic and rhodocladonic acids in *Cladonia rubrotincta*, and the fumarprotocetraric acid complex in *Cladonia coniocraea*), some of which are considered to have antiherbivore properties. Our experiments conducted over a period of 14 days revealed that none of the three lichens was rejected as a food source. When given a choice between two different lichens, mites prefer *C. rubrotincta* over *C. norvegica*, but feed to a similar extent on *C. coniocraea* and *C. rubrotincta*. In experiments only with *C. rubrotincta*, which produces the red chemical compound rhodocladonic acid, a substance with suggested antiherbivore activity, mites surprisingly seem to prefer red-pigmented over unpigmented tissue.

**Supplementary Information:**

The online version contains supplementary material available at 10.1038/s41598-026-54891-5.

## Introduction

Lichens are widely used by various groups of invertebrates as a habitat or food source^1,2^. Smaller animals (e.g., tardigrades, nematodes, mites, and springtails) primarily find refuge in lichens but also have been observed to feed on them^3,4^. Larger animals (e.g., snails, beetles, and butterfly larvae), on the other hand, only tend to consume them^5^. Animal grazing activity can have both positive and negative consequences for lichens. Positive effects include, for example, the spreading of lichen propagules carried on the bodies of small invertebrates and their deposition together with feces^6^. Grazing can induce sturdier lichen growth, which in turn can reduce the risk of damage by wind or other external forces^7^. Coincidentally, the consumption of photobiont cells and physical damage to the lichen tissue caused by feeding often lead to a visible deterioration of lichens^8^, making the negative effects obvious. Lichens have evolved defence mechanisms against invertebrate grazing, such as low concentrations of essential elements reducing food quality, or high concentrations of secondary metabolites^9^. They produce a large array of different secondary metabolites^10,11^, sometimes called lichen substances. Depending on their location in the thallus, lichen substances have been attributed different roles: secondary metabolites occurring mainly in the outer layers of lichens, such as the upper cortex and photobiont layer (e.g. parietin and usnic acid), are hypothesized to protect lichens against solar radiation^12,13^ while compounds produced deeper in the thallus, in the medulla (e.g. vulpinic and pinastric acid) have been suggested to have protective antiherbivore activity^14^. Yet, the role of lichen secondary metabolites in interactions with invertebrates remains ambiguous; some provide protection against specific grazers, but not against all herbivores^3,15,16^. Several studies have demonstrated defensive effects of certain secondary metabolites (e.g. atranorin, lecanoric acid) against grazing invertebrates, especially gastropods^17,18^. Reutimann and Scheidegger^19^ showed that oribatid mites were repelled by fumarprotocetraric acid from the lichen *Cetraria islandica*, but at the same time they were attracted by other secondary metabolites, namely atranorin and norstictic acid, from *Cladonia symphycarpa*.

While controlled feeding experiments of mites and lichens are scarce, lichen-mite interactions have regularly been documented and shown to be manifold. Oribatid mites are frequently associated with lichens^20^ and use them as habitat and food source but also as breeding ground^3,21^. There are numerous accounts of mite infestations harming lichens^8,22,23^, and some authors even consider oribatid mites the chief pests of lichens^24^. That said, the consequences of lichen-mite interactions are rarely obvious, with one notable exception: *Cladonia rubrotincta*, with its characteristic red spots on the thallus, is perhaps the most striking example of an infestation of a lichen with mites. The red color is caused by rhodocladonic acid^25^, a lichen substance commonly occurring in the genus *Cladonia*; however, it is usually restricted only to the reproductive structures^26,27^. Furthermore, the substance is also known from other lichens (e.g. *Mycoblastus sanguinarius*)^28,29^. Rhodocladonic acid production in *Cladonia rubrotincta*, originally identified as *C. norvegica*, was suggested to be a result of grazing mites^30,31^. Recently, we confirmed that the presence of mites correlates with the red coloration, however, not with externally feeding adults, but rather endophagous tunnelling juvenile mites that seem to use tunnels as shelters to complete their development^2^. Various authors^30,32,33^, suggested an antiherbivorous effect of rhodocladonic acid, which should protect the lichen from excessive grazing. However, studies providing a possible explanation for the ecological role of rhodocladonic acid in *Cladonia rubrotincta* are currently lacking. Notably, other morphologically and ecologically similar *Cladonia* species occupying the same habitats and being similarly exposed to grazing mites do not produce this red pigment. In this study, we selected two ecologically similar, yet chemically distinct species (*C. coniocraea* and *C. norvegica*) alongside *C. rubrotincta* to evaluate whether variation in secondary chemistry affects mite grazing. Previous research has shown that the oribatid mite communities associated with these three lichens are largely identical, even across broader geographic scales^2^. While the mites do not appear to discriminate between these lichens in terms of suitable habitat, it remains unclear whether they do so when the lichens are the only available food source. To address this question, we used these three *Cladonia* species as a potential food source, and conducted food choice experiments with adult individuals of *Carabodes areolatus* and *C. marginatus*, which are the most frequently found mite species in these lichens in Europe. Specifically, we aimed to test (1) Do mites distinguish between different lichen species they encounter regularly in their natural habitat? (2) Does the occurrence of certain lichen substances (barbatic, fumarprotocetraic, rhodocladonic acids) influence their feeding behaviour? Answering these questions allows us to gain insights into lichen-mite feeding relationships, the preferences of mites for different lichens and to assess the role of lichen secondary chemistry in lichen-mite interactions.

## Materials and methods

### Lichen and mite species used

A total of four experiments (A: *Cladonia rubrotincta* vs. *C. coniocraea*, tested mite species—*Carabodes marginatus*; B: *Cladonia rubrotincta* vs. *C. norvegica*, tested mite species—*Carabodes marginatus*; C: *Cladonia rubrotincta*, red vs. green parts, tested mite species *Carabodes areolatus*; D: *Cladonia rubrotincta*, red vs. green parts, tested mite species *Carabodes marginatus*) were performed in October 2023 and November 2024. At this time, the name *Cladonia rubrotincta* was not yet formally published, but we were aware, based on molecular results, that it represented a distinct species and treated it accordingly throughout this study.

**Fig. 1:**
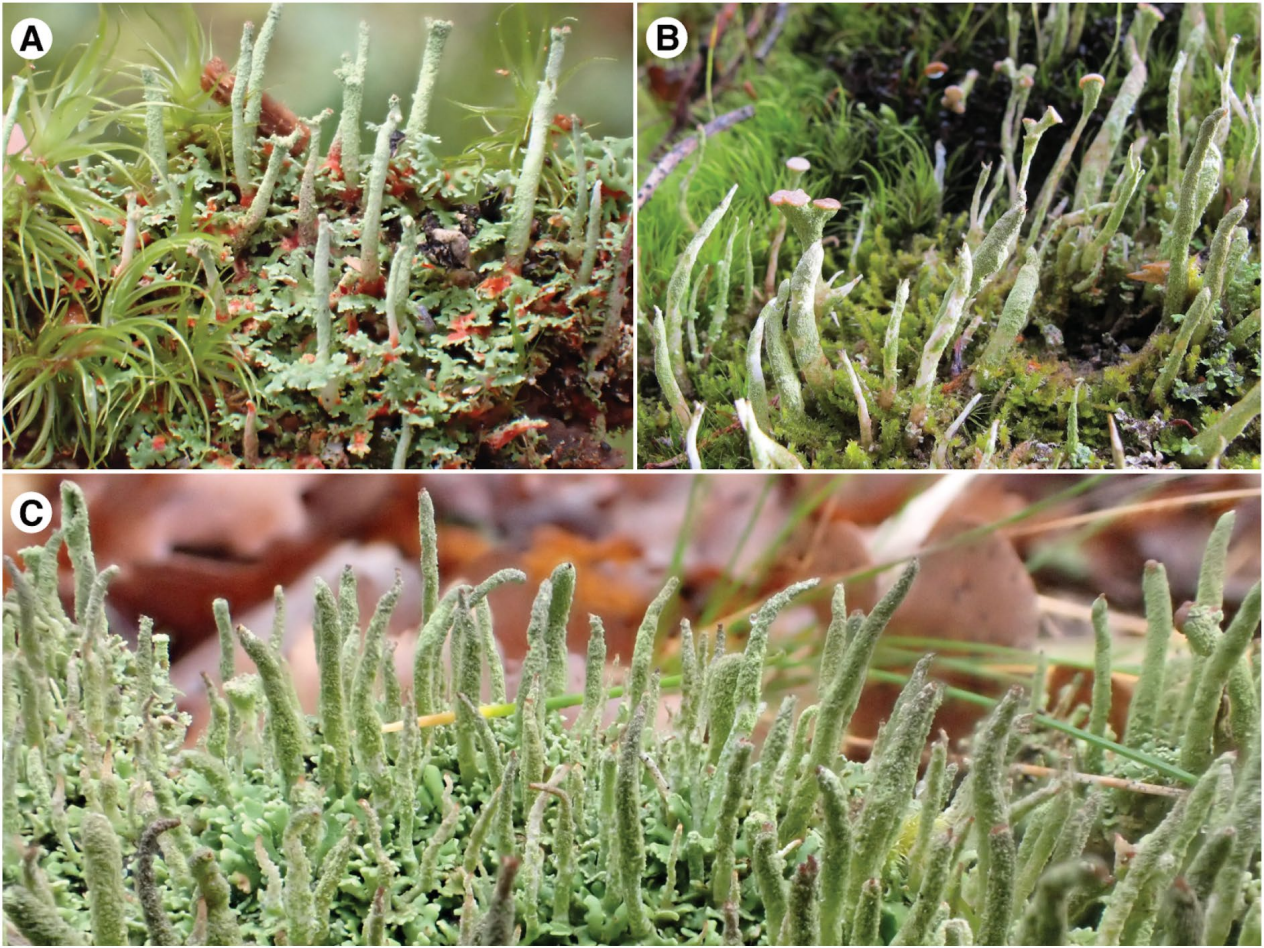
*Cladonia* species used for the experiments. (**A**) *Cladonia rubrotincta* (**B**) *C. norvegica* (Photo:  E. Timdal) (**C**) *C. coniocraea*.

All three *Cladonia* species (Fig. 1) used in the experiments are morphologically and ecologically similar. They are characterized by a dimorphic thallus composed of basal squamules and hollow, sorediate, ascyphose podetia, not exceeding 3 cm in height. *Cladonia coniocraea* typically forms the tallest podetia, whereas *C. rubrotincta* produces the smallest ones (usually not more than 1.5 cm). The three species commonly occur on rotting wood as well as at the bases and on the trunks of trees, especially pine, spruce, and birch^33^. All these habitats are shared with many oribatid mites.

The three species exhibit distinct patterns of secondary metabolite composition. *Cladonia rubrotincta* and *C. norvegica* both contain barbatic and 4-O-demethylbarbatic acids, but *C. rubrotincta* additionally produces rhodocladonic acid and demethyl-rhodocladonic acid in the red spots^25^. *Cladonia coniocraea* contains substances of fumarprotocetraric acid complex^33^, which impart a bitter taste^34^.

Lichen samples used in experiments A and C were collected in Austria (Schöckl mountain; 47.2048925N, 15.4835439E). *Cladonia rubrotincta* used in experiment D was also collected in Austria (Schöckl; 47.2059017N, 15.4819816E). Samples used in experiment B were collected in Norway: *Cladonia rubrotincta* in Enebakk (59.7939028N, 10.9795586E) and *Cladonia norvegica* in Hommelvik (63.3704686N, 10.9076597E), the latter does not occur in Austria. Lichen identification was based on morphology and lichen chemistry using thin-layer chromatography (TLC) according to the standard approach described by Culberson^35^. Lichens used in experiments A, C, and D, collected in Austria, were placed into Berlese-Tullgren funnels on the day of collection and subsequently stored in a dry state at room temperature for 3–5 days, during which the mites were starved. After this short period, lichens were immediately used for the experiments. Lichens from Norway used in experiment B were left in a refrigerator after collection and transported to the Czech Republic in plastic bags within a cooling box. After one week, they were placed into Berlese-Tullgren funnels and then stored in a dry state at room temperature. Samples of *C. rubrotincta* from Norway were stored for 12 months under these conditions, and *C. norvegica* for four months before being used for the feeding experiments with freshly collected mites from Austria. Under these storage conditions, no changes in the profile of secondary metabolites or material degradation are expected, as lichens are adapted to periodic desiccation.

**Fig. 2:**
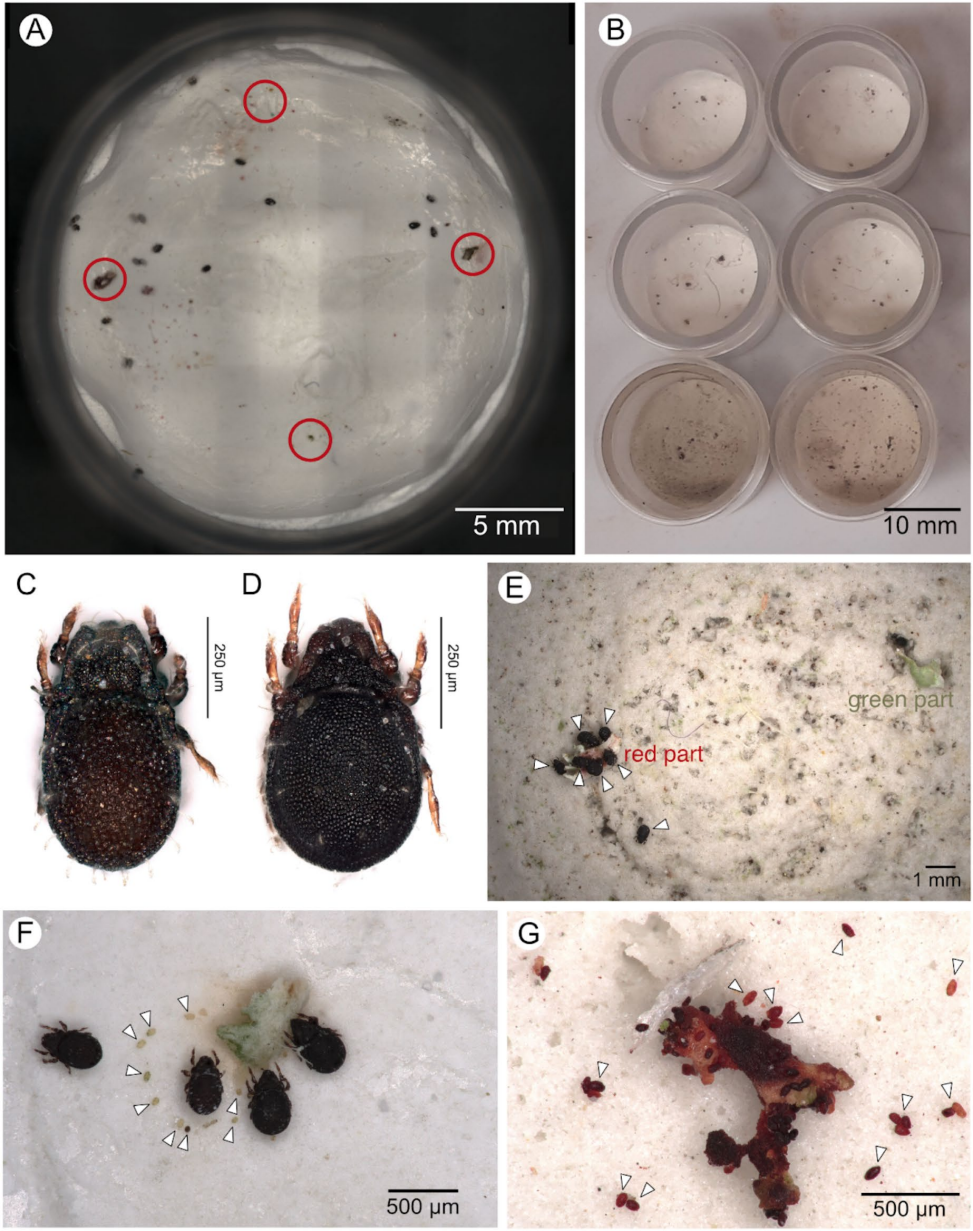
Feeding experiments with *Carabodes* species (**A**) Detailed view of plastic box (the last day—14) with red open circles highlighting offered pieces of lichen (the lichen on top is already consumed); dark spots are mite individuals (**B**) Overview of plastic boxes used for the experiments (**C**) *Carabodes areolatus* specimen photographed in dorsal view (**D**) *Carabodes marginatus* individual in dorsal view (E) Testing red versus green parts of *Cladonia rubrotincta*; white arrowheads pointing to mite individuals aggregating at the red part (day 1) (**F**) Mites feeding on green *Cladonia* lichen thallus; white arrowheads pointing to green fecal pellets (day 8) (**G**) Red lichen thallus of *C. rubrotincta* strongly damaged by feeding; white arrowheads highlighting red fecal pellets deposited by mites (day 8).

The oribatid mite species *Carabodes areolatus* (Fig. 2C) and *C. marginatus* (Fig. 2D) were chosen for the tests because they are the most common mite species to be found in the respective lichens in the studied localities in Austria, Czech Republic and Norway, and their immature stages live in close interaction (inside the thallus) with the lichen *Cladonia rubrotincta*^2^. All mites used in the feeding experiments belonged to the same populations, which were extracted from the lichen samples (*Cladonia rubrotincta*, *C. coniocraea)* and surrounding bark collected on Schöckl mountain (Styria, Austria; see above) with a Berlese-Tullgren funnel immediately after material collection. Mites were identified under a stereomicroscope using Weigmann´s determination key^36^. Before starting the experiment, the mites were starved for 3–5 days. A total of 15 individuals were placed in each feeding box. We only used adult individuals, but we did not determine sex because these species do not show any external sexual dimorphism. Additionally, there are no reports of divergent feeding behaviour between the sexes of microphytophagous oribatid mites, which could have biased our experiments.

We conducted the feeding experiments following the basic setup given in Koukol et al.^3^. For each experiment, six plastic boxes (replicates) lined with plaster at the bottom (2 cm in diameter) were used (Fig. 2B). Each experimental box contained four dry lichen squamule pieces (approximately 1 mm^2^ each) from the collections detailed above, two from each of the two lichen types to be tested (Fig. 2A).

To maintain optimal humidity, the plaster in the plastic boxes was moistened with drops of tap water. The boxes were kept at room temperature (approx. 25 °C).

Mortality was recorded throughout the experiment, but was relatively low in general. Dead mites were replaced with living ones immediately on discovery to maintain a constant density of individuals. Mortality occurred mainly during the initial phase of the experiment (first ~ 7 days), probably because of stabilizing the environmental conditions (e.g., plaster humidity). For more information on experimental interventions and mite mortality, see Supplementary Material (Online Resource 1 ESM1).

### Feeding experiment procedure

In the morning of each day (9:30–12:00), except on weekends, the total number of fecal pellets was counted on the lichen and in its immediate vicinity (within 0.5 cm); ten counts were made over a total of 14 days.

During each count, the moisture content of the plaster was monitored and 3–5 drops of tap water were added if necessary.

A complete overview of the experiments, including the recorded numbers of fecal pellets, is provided in a table in Supplementary Material (Online Resource 1 ESM1). In some boxes, the number of fecal pellets slightly decreased due to the occasional displacement of the lichen pieces on which pellets were deposited, rare mold growth, but possibly also due to the mites occasionally feeding on fecal pellets, as observed in the study of Koukol et al.^37^. The experiments were terminated after 14 days.

### Statistical analysis

For statistical analyses, the table containing the test results was simplified. Fecal pellets found directly on the lichen and in its close vicinity were counted and summed. Pellets on both pieces of the same lichen species were also summed. Boxes in which no feeding (less than 10 pellets in 14 days) occurred were excluded from the analysis (see Supplementary ESM 1). This resulted in three removed datapoints for experiment A (three boxes remaining), one removed datapoint for experiment B (five boxes remaining) and two removed datapoints for experiment C (four boxes remaining). No data points needed to be removed from experiment D (six boxes).

**Fig. 3:**
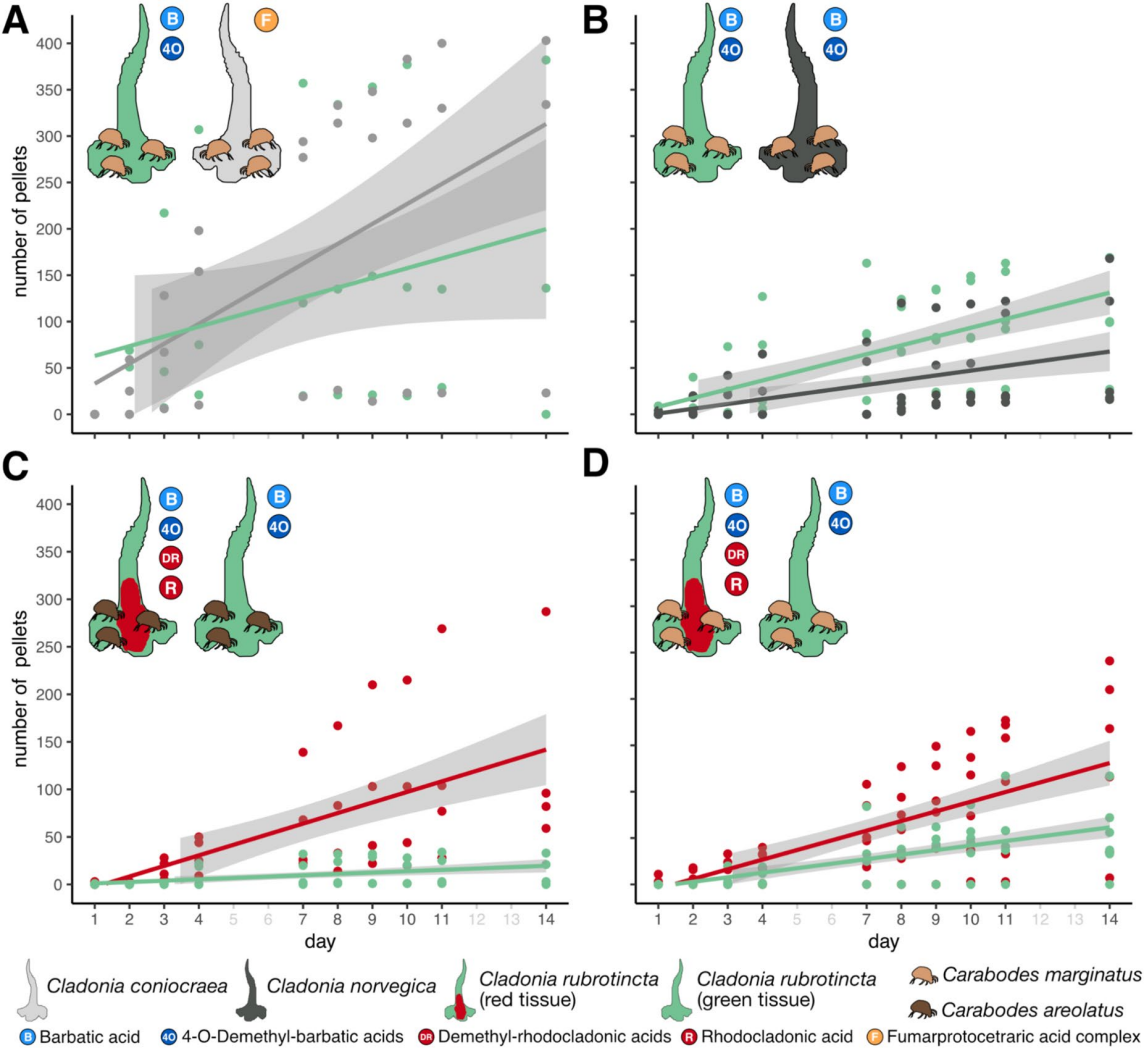
Results of food choice experiments. Individual points indicate the number of mite fecal pellets recorded at specific times. Different colors indicate different lichens or different lichen tissue. Days with greyed out numbers are days on which fecal pellets were not counted (weekends). Small, colored circles beside lichen silhouettes inside individual plots refer to different lichen substances produced by the respective lichen or tissue. Trend lines indicate smoothed conditional means of fecal pellet number; grey areas around trend lines are 95% confidence intervals. Panels indicate different experimental setups: (**A**) *Cladonia rubrotincta* (green tissue) against *C. coniocraea* with *Carabodes marginatus* (**B**) *Cladonia rubrotincta* (green tissue) against *C. norvegica* with *Carabodes marginatus* (**C**) *Cladonia rubrotincta* (red tissue) against *C. rubrotincta* (green tissue) with *Carabodes areolatus* (**D**) *Cladonia rubrotincta* (red tissue) against *C. rubrotincta* (green tissue) with *Carabodes marginatus*.

Following Koukol et al.^37^, we chose Generalized Linear Mixed Effects Models (GLMM) for data processing. GLMMs allow to test effects of categorical variables (mite species, lichen species) and continuous variables (time period) on the dependent variable (counts of fecal pellets) in one model. We selected mite species, lichen part/species and time period (days) as fixed effects. Variability between individual replicates (boxes) was considered a random effect. A Poisson probability distribution was used. The Omnibus test based on Chi-square was used for analyses of fixed effects. Odd Ratios (expB) were used as measures of effect size. Due to the significance of the effects, this analysis was followed by post-hoc tests using Bonferroni correction of *p*-values ​​for detailed multiple comparisons of individual groups. The results were graphically displayed in scatter plots with linear predictor lines and standard errors of means as measures of variability. Analyses were performed in the desktop version of the Jamovi program (Version 2.6)^38,39^ using the GAMLj package^40^. Scatter plots (Fig. 3) were generated with R v4.4.2^38^ using ggplot2 v3.5.1 and patchwork v1.2.0.

In this study, mites found on individual lichen pieces within each experiment were also counted. These counts showed the same trend compared to fecal pellets data, but were less precise, because the mites were disturbed during the counting and some of them moved around. Therefore, the mite counts were not used in further analysis. Counting fecal pellets is an established method in microarthropod feeding experiments and proved to be reliable for determining feeding preferences. We used these fecal pellet counts as the primary measure of feeding activity because they directly reflect ingestion rather than mere presence of mites, and pellets were consistently spatially associated with the corresponding lichen material (i.e. red pellets were found only on red-pigmented parts and green pellets only on green parts).

## Results

### General outcomes

Statistical analysis of fecal pellet data revealed three main results (Table 1): First, all lichen material was readily consumed by the investigated mites, regardless of the lichen substance profiles of the used lichen food offerings. Second, *Carabodes marginatus* preferred *Cladonia coniocraea* over *C. rubrotincta,* and it preferred *Cladonia rubrotincta* over *C. norvegica*. Third, when mites were presented with both red-spotted (rhodocladonic acid-containing) and green (rhodocladonic acid-absent) tissue of *C. rubrotincta*, both investigated mite species seemed to prefer red-spotted lichen material.

**Table 1:** Summary of results of the Generalized mixed model analyses (GLMM) for all four feeding preference experiments (A–D). The preferred lichen in each experiment is in bold. Detailed results are available in Supplementary Material (Online Resource 2 ESM2).

Experiment	Fixed effects		Replicates	χ^2^	Effect size	p-value
A	Mite	*Carabodes marginatus*	3			
	Lichen	*C. rubrotincta* vs ***C. coniocraea***		67.9	0.838	< .001
	Day			628.2	1.623	< .001
B	Mite	*Carabodes marginatus*	5			
	Lichen	***C. rubrotincta*** vs *C. norvegica*		540.1	2.05	< .001
	Day			26.0	1.17	< .001
C	Mite	*Carabodes areolatus*	4			
	Lichen	***C. rubrotincta*** -red vs *C. rubrotincta-*green		1213.4	0.150	< .001
	Day			24.3	1.190	< .001
D	Mite	*Carabodes marginatus*	6			
	Lichen	***C. rubrotincta-*** red vs *C. rubrotincta-*green		621.7	0.470	< .001
	Day			39.5	1.139	< .001

### *Cladonia* lichens as a food source

Our experiments revealed that all three offered lichen species are readily consumed by mites. This is apparent by the constant increase of fecal pellets over the course of 14 days in all experiments (Fig. 3). This increase is independent of the used mite species or the provided food choices. However, we were able to identify preferences depending on different food offerings.

### Feeding preferences using different *Cladonia* lichen species

To test whether mites prefer one lichen food source over another, when both can co-occur in the same habitat, we provided tissue of the ecologically similar *C. norvegica* and *C. rubrotincta* as alternative food sources. *Cladonia coniocraea* is more distantly related; it belongs to the clade *Cladonia*, whereas *C. norvegica* is the sister species of *C. rubrotincta* inside the clade Ochroleucae^25^. We performed these experiments only with *Carabodes marginatus,* since we were unable to obtain a sufficient number of *C. areolatus* mites for all experiment replicates (4 experiments with 6 replicates and 15 specimens in each replicate would require 360 mite individuals).

We found that *Carabodes marginatus* significantly (*p* < 0.001) preferred the ecologically similar, yet phylogenetically and chemically different *Cladonia coniocraea* over *C. rubrotincta* (see Table 1; experiment A). However, in one of the three replicates, we observed the opposite preference for *C. rubrotincta* over *C. coniocraea*. This resulted in a high variability of data and overlapping standard deviations of regression lines (Fig. 3A).

We found that *Carabodes marginatus* significantly (*p* < 0.001) preferred green *Cladonia rubrotincta* tissue over that of its sister species *C. norvegica* (see Table 1; experiment B). The GLMM analysis (see Table 1; experiment B) revealed significant effects (*p* < 0.001) of the fixed factors—lichen and day. The total number of fecal pellets increased significantly with each successive day of the experiment. *Cladonia rubrotincta* was the preferred lichen species, as is also illustrated in the plot below (Fig. 3B).

### *Cladonia rubrotincta* as a sole food source, red versus green parts

When offering *Cladonia rubrotincta* tissue with and without rhodocladonic acid (indicated by red spots), both investigated *Carabodes* species seemed to prefer lichen material with rhodocladonic acid (Figs. 2E and 3C, D).

Results of the GLMM analysis (Table 1; experiment C, D) showed significant differences (*p* < 0.001) of individual fixed effects (mite species, lichen part, time period) for both mite species. The total number of fecal pellets increased significantly with each subsequent day of the experiment, indicating continued feeding over the whole duration of the experiment (Fig. 3 C, D).

In order to compare possible variations in preferences of *Carabodes areolatus* and *Carabodes marginatus* for red vs. green parts of *Cladonia rubrotincta* we performed an additional GLMM analysis. For this, we combined data from both mite species into a single model, followed by post-hoc tests (see Supplementary Material—Online Resource 2, ESM 2). The interaction between mite species and lichen part was significant (*p* < 0.001), and preference for red parts of the lichen was more pronounced in *Carabodes areolatus* (*p* < 0.001, large effect size, expB = 6.658) than in *C. marginatus* (*p* < 0.001, medium effect size , expB = 2.126).

Our observations also showed that the fecal pellets of mites were colored according to the part of the lichen that the mites consumed. White (transparent), green, and red pellets were observed (see Fig. 2F, G).

## Discussion

### Mites consume all offered lichen material but show certain preferences

The majority of oribatid mites can be divided into three main feeding guilds: macrophytophagous feeders (feeding on litter, wood and pollen), microphytophagous grazers (feeding on fungi, algae and bacteria) and panphytophagous browsers (feeding on diverse plants and humus)^41^, whereas these guilds can be further subdivided into at least five different groups based on their enzyme equipment^42^. Species of the genus *Carabodes* are classified in a group of opportunistic herbo-fungivores, which are known to include lichenivorous grazers^42^. *Carabodes* mites are commonly found in lichens^43,44^, and the here investigated *C. marginatus* and *C. areolatus* were the two most abundant species previously found in the Austrian, Norwegian and Czech material of *Cladonia coniocraea*, *C. norvegica* and *C. rubrotincta* we investigated^2^. We recently reported that juveniles of these *Carabodes* species burrow tunnels in *Cladonia rubrotincta* lichen thalli and feed on them from inside. This activity is correlated with the production of rhodocladonic acid^2,25^. Despite having endophagous juveniles, the adults of both investigated species have only rarely been observed to feed on *Cladonia* spp., according to a literature review of lichen-mite interactions^3^. Instead, they were reported mainly from other habitats like mosses, soil and deadwood^36^. Accordingly, both species show identical ecologies and so far no species-specific feeding strategies have been observed . Thus, it remained unclear if adult mites really use these lichens as a food source. Oribatid mites possess chemosensitive organs on their pedipalps which they can use for smelling and tasting^36^. We observed how the mites in our experiments walked around using their pedipalps probing the found substrates before they started feeding, which indicated that they actively chose their food. The present feeding experiments clearly highlight that adults of *Carabodes marginatus* feed on all three offered lichen species, albeit with certain preferences when different lichens were offered together. Which factors actually influence mite grazing on lichens, here and in previous studies? It remains challenging to deduce generalizable similarities across different tested mites, lichens and experimental conditions of previous studies. That said, the main factor brought forward to explain feeding pressure by mites are differences in lichen secondary metabolite composition^19,45,46^.

In our first pairwise food-choice test, we found that *C. coniocraea* is preferred over *C. rubrotincta* (Fig. 3A), but in one box we could observe the opposite effect, which caused large variability in our results. Nevertheless, the general preference for *C. coniocraea* is somewhat unexpected as it produces fumarprotocetraric acid, which has previously been reported to deter invertebrate grazers, including oribatid mites^19,46^. Reutimann and Scheidegger^19^ reported that oribatid mites, including a *Carabodes* species, were repelled by the fumarprotocetraric acid present in the lichen *Cetraria islandica*. However, *Cetraria islandica* also contains protolichenesterinic acid, and it remains unclear whether fumarprotocetraric acid or this additional substance alone or together are responsible for a repellent effect. If fumarprotocetraric acid indeed has the potential to deter grazing invertebrates, why did we not see this effect in our experiments? Lichen substance concentrations can vary under different environmental conditions in the same species^47^. Also, individual lichen parts of single thalli can differ in their substance profile ^48^, a trait so conserved that it is also used for lichen identification. Grazing on tissues differing in lichen substance composition or concentration offers an explanation for the diverging findings of previous studies and ours. It could also explain why in one of our boxes *C. rubrotincta* was preferred over *C. coniocraea*, perhaps the used lichen piece in this specific box differed in substance concentration or profile from the other lichen pieces used in the same experiment. Meier et al.^6^, for example, showed that mites avoided the lower cortex of the lichen *Xanthoria parietina*, and also snails differentially feed on *Lobaria pulmonaria* following the distribution of secondary metabolites within the thallus^49^. Although not explicitly tested here, during our daily visual inspections of the feeding boxes, we observed that all thallus parts of *C. coniocraea* were readily consumed. This leaves the possibility that either a low concentration or different localization of fumarprotocetraric acid in the provided lichen material explains our findings. However, Asplund et al.^46^ demonstrated that the abundance of oribatid mites in some lichens was only little affected by removing secondary compounds and that there can be opposing responses of different oribatid mites to these compounds. Accordingly, fumarprotocetraric acid could successfully repel certain lichenivorous invertebrates, while the here used *Carabodes marginatus* remains unaffected.

Apart from fumarprotocetraric acid, the other main difference in the secondary metabolite profiles of all three tested lichen species concerns the presence of substances from the barbatic acid complex. Barbatic acid and related 4-O-demethylbarbatic acid^25^ are present in *Cladonia rubrotincta* and *C. norvegica*, but they are missing in *Cladonia coniocraea*. Barbatic acid has been shown to have antimicrobial activity^50^, but an antiherbivore activity has, to our knowledge, not been suggested. In our case, the lack of barbatic acid and its derivatives could explain why mites readily used *C. coniocraea* as a food source and why they seemed to prefer it over *C. rubrotincta* (Fig. 3A).

However, mites also feed on the two barbatic- and 4-O-demethylbarbatic acid-containing *Cladonia* species (Fig. 3B–D), which suggests only a mild repellent effect, at least against *Carabodes* mites. We suggest investigating the antiherbivore role of these substances in future experiments.

Besides the absence or presence of secondary metabolites, thallus structure has also been suggested to explain the feeding behavior of mites in previous work^19^, and lichen growth-forms and physiological traits have been shown to be responsible for changes in mite community composition^51^. As mentioned above, in our experiments, mites fed equally on different lichen parts. However, while all tested *Cladonia* lichens exhibit very similar thallus structure, except for the larger size of squamules and podetia in *C. coniocraea*, it is impossible to rule out that other morphological differences invisible to us but detectable by the mites are responsible for the observed preferences.

Another reason why *Carabodes marginatus* prefers one lichen over another could be differences in their respective nutrient content. Invertebrate community composition has been shown to be influenced by differences in nitrogen and phosphorous content in lichens^51^. Baur et al.^15^ found that molluscs prefer lichens that enable juveniles to grow fastest (which were not always those with the lowest content of secondary metabolites). It is possible that *C. coniocraea* provides a better-suited nutrient profile and is therefore preferred. In contrast, *Carabodes marginatus* juveniles have only been found tunnelling and feeding on *C. rubrotincta*^2^ and not in *C. coniocraea.* Consequently, it can be assumed that *C. rubrotincta* provides better conditions for the development of juvenile mites. If these better conditions are correlated with nutrient content, physical protection or anything else is yet unknown and will have to be investigated in future experiments in detail.

We also gave *Carabodes marginatus* the choice between *Cladonia rubrotincta* and *C. norvegica* (Fig. 3B), in which case they preferred to feed on *Cladonia rubrotincta*. This is again unexpected, since these two lichens are morphologically almost identical. *Cladonia rubrotincta* and *C. norvegica* also show the same composition of secondary metabolites, except for the rhodocladonic acid, which is only produced in definite red colored areas of *C. rubrotincta*^25^, but in this experiment, we specifically avoided these areas to limit the influence of rhodocladonic acid on the outcome. This leaves only differences in the concentration of secondary metabolites or in nutrient content as possible causes for the observed feeding preference.

### The role of rhodocladonic acid

The effects of rhodocladonic acid on animals (including mites) have been rarely studied. Some studies^52,53^ highlight its herbicidal and allelopathic properties, others^13^ suggest that lichen substances in fruiting bodies ' protect the reproductive structures against UV-radiation and infection by bacteria, fungi and other organisms. In *Cladonia rubrotincta,* the production of rhodocladonic acid in certain parts of the thalli was considered a direct response to mite feeding activity, since it was first described by Timdal^30^ and accordingly considered a defensive response against mite grazing. Our recent studies^2,25^ confirmed that red parts of *C. rubrotincta* containing rhodocladonic acid were associated with the feeding activity of juvenile mites, but a direct repellent effect of the substance could not be demonstrated. The present results now extend this to adult mites, as thallus parts containing rhodocladonic acid were preferred by adult *Carabodes marginatus* and *C. areolatus* (Fig. 3C, D). This could indicate that rhodocladonic acid, in fact, provides certain benefits to the mites during feeding. For instance, this substance could aid mites to digest the lichen more efficiently, or it can simultaneously increase the content of elements beneficial to the mites. A visual attraction of mites by the red color of this compound is rather unlikely. Mites lack color vision (in fact, most do not see at all; some oribatids possess simple ocelli or so-called ‘lenticuli’ that only allow the distinction between light and darkness^54^). Alternatively, mite attraction may be facilitated by volatile compounds released by the lichen. *Cladonia* lichens have been shown to produce volatile compounds^55^, however, their ecological function remains poorly understood. Differences in volatile compound profiles have also been reported among *Cladonia* species^55^, suggesting species-specific chemical signals.

It is also possible that the adult mites were actually not attracted by any lichen secondary metabolites produced inside the red tissue of *C. rubrotincta*, but by some chemical compounds produced by endophagous mite juveniles, signalling a suitable breeding ground, which is pure conjecture right now. The red tissue used in the experiments did not contain any juveniles and it would not make much sense if adults feed on the shelter of their own juveniles. It is yet unknown how adults choose the breeding ground and how specifically they lay their eggs into the lichen tissue, once this question is answered, we might also know if chemical compounds of juveniles play a role in this respect.

However, our results also do not rule out a negative yet more subtle effect of rhodocladonic acid or other lichen substances. Lichen substances may affect feeding mites after ingestion as lichen material passes through the mites’ digestive tract. Meier et al.^6^ noted that the color of fecal pellets matches the color of ingested material, and we could also observe this phenomenon. The color of fecal pellets differed depending on the parts consumed: red in the case of feeding on the red parts of *Cladonia rubrotincta* (Fig. 2G), green when feeding on the photobiont layer of the green parts of the same lichen (Fig. 2F), and white when feeding on the medulla. The red color of fecal pellets indicates that a sufficient amount of rhodocladonic acid remains intact during the passage through the digestive tract of oribatid mites. Yet, even small metabolized quantities of rhodocladonic acid and other lichen substances could still influence mite behavior, metabolism or their associated microorganisms. To better understand the role of rhodocladonic acid (and other lichen substances) in the interaction with oribatid mites, their effects after ingestion should be examined further in adults as well as juvenile mites.

Few studies deal with mite-lichen feeding relationships, and they all were performed under somewhat artificial conditions, including ours. Often, lichen substances were completely removed by treatment with acetone^19,46^, or experiments were performed under laboratory conditions^19^ or involved transplanting of lichen material^46^. In the future, more comprehensive studies, also under natural conditions, are needed to understand the complex role of lichen secondary metabolites and their possible functions in animal-lichen interactions.

### Effects of mite feeding

Oribatid mites are tiny animals and, despite some cases where the opposite was reported^8,22,23^, may not generally impact lichen fitness under natural conditions. Rather, we suggest that the antiherbivore potential of certain lichen substances, like fumarprotocetraric acid, evolved probably as a response against larger grazers but not against the tiniest. Reindeer, for example, have been reported to avoid fumarprotocetraric acid containing *Cladonia* species^34^, although there have also been contrasting reports suggesting that fumarprotocetraric acid containing lichens make up a large part of the winter diet of deer due to high nutrient availability (e.g. *Cladonia arbuscula*, *C. gracilis* and *Cetraria islandica*^56^). Nevertheless, it is clear that animals at the mega- and macro-scale (i.e. vertebrates, slugs, snails, beetles) can cause serious damage to the lichens by feeding, whereas smaller animals at the meso- and micro-scale (i.e. mites, springtails, tardigrades, nematodes) will cause negligible damage through their grazing activity, unless they occur in extreme masses. Mites have been shown to promote dispersal of lichens by spreading lichen propagules either with their fecal pellets^57,58^ or on their external body surfaces^59^. Moreover, herbivores, mites included, can disrupt the cortex of lichens, uncovering algal cells which then can serve as a photobiont source for other lichens^60^. Although studies investigating this topic are scarce, it can be assumed that the obvious positive effect of aided dispersal can counteract the evolution of strong chemical or mechanical defence mechanisms by lichens.

## Conclusion

The present study highlights that three ecologically similar lichens with different lichen secondary substance profiles are readily consumed by oribatid mites. We argue that the often-suggested defensive role of some lichen substances may be more nuanced than previously assumed. While feeding behavior was similar on lichens regardless of their different chemical profiles, we hypothesize that potential negative effects by lichen substances may have evolved to deter other, larger animals than oribatid mites. Should a negative effect of lichen substances on mites exist, then it is probably more subtle and will require additional experiments monitoring mite behavior; and gene expression during consumption of different lichen material.

## Supplementary Information

Supplementary Information.

## Data Availability

All data produced from this study are provided in this manuscript.
