# Supplementary material for: Oribatid mites like lichens—feeding preferences among three Cladonia species with different chemistry
Source: Sci Rep. 2026 May 31;16:24894. doi: 10.1038/s41598-026-54891-5 (PMC13458371; doi:10.1038/s41598-026-54891-5)
Supplement: Supplementary file 1 [file 41598_2026_54891_MOESM1_ESM.pdf]

Oribatid mites like lichens - feeding preferences among three *Cladonia* species with different chemistry

Věra Vtípilová, Tobias Pfingstl*, Heda Ghlimová, Jan Mourek, Jana Steinová and Philipp Resl
*Corresponding author: Tobias Pfingstl, e-mail: tobias.pfingstl@uni-graz.at, tel: +43 3163805611

Online Resource 1: Daily fecal pellets counts and experimental interventions

Experiment A: *Cladonia rubrotincta* vs. *C. coniocraea*, tested mite species - *Carabodes marginatus*

Box ID	DAY 0		DAY 1		DAY 2		DAY 3		DAY 4		DAY 7		DAY 8		DAY 9		DAY 10		DAY 11		DAY 14	
	conio	rubro	conio	rubro	conio	rubro	conio	rubro	conio	rubro	conio	rubro	conio	rubro	conio	rubro	conio	rubro	conio	rubro	conio	rubro
26MA	0	0	0	0	0	2	2	1	2	4	2	9	0	3	0	5	2	5	2	5	2	5
27MA	0	0	0	1	0	0	3	9	4	0	4	0	4	4	4	5	3	5	3	5	3	4
28MA	0	0	0	0	0	0	4	4	11	1	5	5	0	10	3	4	3	4	2	4	2	4
29MA	0	0	0	0	59	51	128	46	198	75	277	120	333	135	298	149	314	137	330	135	334	136
30MA	0	0	0	0	25	0	67	7	154	21	294	20	314	21	348	21	383	20	400	29	403	0
31MA	0	0	0	0	0	69	6	217	10	307	19	357	26	334	14	363	23	377	23	447	23	382

Experiment B: *Cladonia rubrotincta* vs. *C. norvegica*, tested mite species - *Carabodes marginatus*

Box ID	DAY 0		DAY 1		DAY 2		DAY 3		DAY 4		DAY 7		DAY 8		DAY 9		DAY 10		DAY 11		DAY 14	
	norve	rubro	norve	rubro	norve	rubro	norve	rubro	norve	rubro	norve	rubro	norve	rubro	norve	rubro	norve	rubro	norve	rubro	norve	rubro
25	0	0	4	9	**20	40	42*	73*	**65	127*	**57	163	18	116	21	134	21	149	18	154	18	169
26	0	0	0	0	**0	0	0*	0*	**0	0	**0*	37	3	67	10	83	13	82	13	92	16	99
27	0	0	2	0	**2	0	2*	0*	2	0	**2*	0*	0	0	0	0	0	0	0	0	0	0
28	0	0	0	0	**0	0	0*	2*	**0	6	**0*	15	6	17	12	24	19	24	20	27	24	27
29	0	0	0	0	**18	7	21*	25*	**25	75	78	86	120	68	115	80	119	83	122	100	122	100
30	0	0	0	0	**2	0	0*	0*	**0	14	**0*	87	12	124	53	135	55	144	109	163	168	169

Experiment C: *Cladonia rubrotincta*, red vs. green parts, tested mite species *Carabodes areolatus*																						
Box ID	DAY 0		DAY 1		DAY 2		DAY 3		DAY 4		DAY 7		DAY 8		DAY 9		DAY 10		DAY 11		DAY 14	
	green	red	green	red	green	red	green	red	green	red	green	red	green	red	green	red	green	red	green	red	green	red
1A	0	0	0	3	0	3	0	22	0	44	2	68	2	83	**1	103	1	103	3	104	3	96
2A	0	0	0	0	0	0	1	5*	22	9	32*	26*	32	33*	32	41	20	44	34	77	21+	82
3A	0	0	0	0	0	0	1	11	19*	25	20*	22	24	14	29	22	28	20	25	28	33	59
4A	0	0	1	0	0	2	0	28	0	50	0*	139*	1	167	1	210	1	215	1	269	0	287+
5A	0	0	1	0	0	0	0	0	0	0	0	3	0	3	0	0	0	0	0	0	0	0
6A	0	0	0	0	0	2	0	0	0	0	0	0	0	0	0	0	0	1	0	1	0	0

Experiment D: *Cladonia rubrotincta*, red vs. green parts, tested mite species *Carabodes marginatus*																						
Box ID	DAY 0		DAY 1		DAY 2		DAY 3		DAY 4		DAY 7		DAY 8		DAY 9		DAY 10		DAY 11		DAY 14	
	green	red	green	red	green	red	green	red	green	red	green	red	green	red	green	red	green	red	green	red	green	red
9	0	0	0	11	3	16	20*	24*	19	32	**25	47	35	75	49	90	50	137	58	177	72	241
10	0	0	0	0	**0	5	2*	16*	**12	18	**84	85	62	94	62	128	86	118	117	158	117	168
11	0	0	0	1	0	0	0*	0*	**1	0	**25	0*	35	0	43	0	57	3	41+	3	37+	7
12	0	0	0	3	**2	18	3*	33*	**12	33	33	19	31	28	43	39	45	37	58	33+	56	35+
13	0	0	0	0	1	6	1*	16*	**1	40	**0*	108	0	127	0	149	0	165	0	172	0	210
14	0	0	0	0	**0	0	0*	1*	0	1	**26*	51	38	59	41	78	34	74	37	111	33	116

Legend:

*	Lichen pieces were replaced or added (in case of mold overgrowth or consummated lichen)	green	Green tissue of *C. rubrotincta*
**	Replacement of dead mites with live individuals (approximately 2 - 3 individuals)	red	Red tissue of *C. rubrotincta*
+	Mold contamination was observed	conio	*Cladonia coniocraea*
		rubro	*Cladonia rubrotincta*
Box ID	Excluded from analysis	norve	*Cladonia norvegica*

Online Resource 2: Complete results of the Generalized Mixed Model Analyses of the feeding preference experiemnts A-D. Analyses were performed in the Jamovi program (R Core Team 2021; The Jamovi Project 2022) using the GAMLj package (Gallucci 2019).

Experiment A - *Carabodes marginatus* feeding on *Cladonia rubrotincta* vs. *Cladonia coniocraea*, performer in 3 replicates.

Generalized Mixed Model

Model Info

Info	Value	Comment
Model Type	Poisson	Model for count data
Call	glm	pellets ~ 1 + lichen + day + (0 + day | box)
Link function	log	Coefficients are in the log(y) scale
Distribution	Poisson	Model for count data
LogLikel.	-3903.656	Unconditional Log-Likelihood
-2*LogLikel.	7807.311	Unconditional absolute deviance
Deviance	7475.998	Conditional relative deviance
R-squared	0.967	Marginal
R-squared	0.970	Conditional
AIC	7815.310	Less is better
BIC	7823.621	Less is better
Residual DF	55.000	
Chi-squared/DF	119.652	Overdispersion indicator
Converged	yes	
Optimizer	bobyqa	

Model Results

Fixed Effect Omnibus tests

	X^2	df	p
**lichen**	67.9	1.00	<.001
**day**	628.3	1.00	<.001

Fixed Effects Parameter Estimates

					95% Exp(B) Confidence Interval			
**Names**	**Effect**	**Estimate**	**SE**	**exp(B)**	**Lower**	**Upper**	**z**	**p**
(Intercept)	(Intercept)	4.882	0.0119	131.918	128.867	135.042	408.88	<.001
lichen	C.rubrotincta vs. C. **coniocraea***	-0.177	0.0215	0.838	0.803	0.874	-8.24	<.001
day	day	0.484	0.0193	1.623	1.563	1.686	25.06	<.001

* Preferred lichen printed in bold.

Random Components

Groups	Name	SD	Variance	ICC
**replicate**	day	0.0272	7.41e-4	
**Residuals**		1.0000	1.00	.

Note. Number of Obs: 59 , groups: 3 replicates

Experiment B - *Carabodes marginatus* feeding on *Cladonia rubrotincta* vs. *Cladonia norvegica*, performed in 5 replicates.

Generalized Mixed Model

Model Info

Info	Value	Comment
Model Type	Poisson	Model for count data
Call	glm	pellets ~ 1 + lichen + day + (0 + day | box)
Link function	log	Coefficients are in the log(y) scale
Distribution	Poisson	Model for count data
LogLikel.	-1803.612	Unconditional Log-Likelihood
-2*LogLikel.	3607.225	Unconditional absolute deviance
Deviance	3167.382	Conditional relative deviance
R-squared	0.840	Marginal
R-squared	0.958	Conditional
AIC	3615.220	Less is better
BIC	3625.645	Less is better
Residual DF	96.000	
Chi-squared/DF	32.788	Overdispersion indicator
Converged	yes	
Optimizer	bobyqa	

Model Results

Fixed Effect Omnibus tests

	X^2	df	p
**lichen**	540.1	1.00	<.001
**day**	26.0	1.00	<.001

Fixed Effects Parameter Estimates

					95% Exp(B) Confidence Interval			
**Names**	**Effect**	**Estimate**	**SE**	**exp(B)**	**Lower**	**Upper**	**z**	**p**

(Intercept)	(Intercept)	3.560	0.0184	35.15	33.90	36.44	193.03	<.001
lichen	**C. rubrotincta** vs. C. norvegica*	0.718	0.0309	2.05	1.93	2.18	23.24	<.001
day	day	0.154	0.0303	1.17	1.10	1.24	5.10	<.001

* Preferred lichen printed in bold.

Random Components

**Groups**	**Name**	**SD**	**Variance**	**ICC**
**replicate**	day	0.0671	0.00450	
**Residuals**		1.0000	1.00000	.

Note. Number of Obs: 100 , groups: 5 replicates

Experiment C - *Carabodes areolatus* feeding on green vs. red parts of *Cladonia rubrotincta*, performed in 4 replicates.

Generalized Mixed Model

Model Info

Info	Value	Comment
Model Type	Poisson	Model for count data
Call	glm	pellets ~ 1 + lichen + day + (0 + day | box)
Link function	log	Coefficients are in the log(y) scale
Distribution	Poisson	Model for count data
LogLikel.	-1110.387	Unconditional Log-Likelihood
-2*LogLikel.	2220.774	Unconditional absolute deviance
Deviance	1906.283	Conditional relative deviance
R-squared	0.925	Marginal
R-squared	0.976	Conditional
AIC	2228.770	Less is better
BIC	2238.497	Less is better
Residual DF	80.000	
Chi-squared/DF	24.444	Overdispersion indicator
Converged	yes	
Optimizer	bobyqa	

Model Results

Fixed Effect Omnibus tests

	X^2	df	p
**lichen**	1213.4	1.00	<.001
**day**	24.3	1.00	<.001

Fixed Effects Parameter Estimates

	95% Exp(B) Confidence Interval

Names	Effect	Estimate	SE	exp(B)	Lower	Upper	z	p
(Intercept)	(Intercept)	2.847	0.0311	17.235	16.216	18.318	91.57	<.001
lichen	C. rubrotincta - green vs. **C. rubrotincta - red***	-1.896	0.0544	0.150	0.135	0.167	-34.83	<.001
day	day	0.174	0.0353	1.190	1.111	1.276	4.93	<.001

* Preferred lichen printed in bold.

Random Components

Groups	Name	SD	Variance	ICC
**replicate**	day	0.0699	0.00489	
**Residuals**		1.0000	1.00000	.

Note. Number of Obs: 84 , groups: 4 replicates

Experiment D - *Carabodes marginatus* feeding on green vs. red parts of *Cladonia rubrotincta*, performed in 6 replicates.

Generalized Mixed Model

Model Info

Info	Value	Comment
Model Type	Poisson	Model for count data
Call	glm	pellets ~ 1 + lichen + day + (0 + day | box)
Link function	log	Coefficients are in the log(y) scale
Distribution	Poisson	Model for count data
LogLikel.	-1717.990	Unconditional Log-Likelihood
-2*LogLikel.	3435.979	Unconditional absolute deviance
Deviance	2925.411	Conditional relative deviance
R-squared	0.859	Marginal
R-squared	0.961	Conditional
AIC	3443.980	Less is better
BIC	3455.129	Less is better
Residual DF	116.000	
Chi-squared/DF	20.811	Overdispersion indicator
Converged	yes	
Optimizer	bobyqa	

Model Results

Fixed Effect Omnibus tests

	X^2	df	p
**lichen**	621.7	1.00	<.001
**day**	39.5	1.00	<.001

Fixed Effects Parameter Estimates

					95% Exp(B) Confidence Interval			
**Names**	**Effect**	**Estimate**	**SE**	**exp(B)**	**Lower**	**Upper**	**z**	**p**

(Intercept)	(Intercept)	3.325	0.0193	27.803	26.771	28.874	172.36	<.001
lichen1	C. rubrotincta - green vs. **C. rubrotincta - red** *s	-0.754	0.0302	0.470	0.443	0.499	-24.93	<.001
day	day	0.189	0.0300	1.208	1.139	1.281	6.28	<.001

* Preferred lichen printed in bold.

Random Components

**Groups**	**Name**	**SD**	**Variance**	**ICC**
**replicate**	day	0.0729	0.00532	
**Residuals**		1.0000	1.00000	**.**

Note. Number of Obs: 120 , groups: 6 replicates

Experiment C+D – combined model for both mite species *Carabodes areolatus* (4 replicates) and *Carabodes marginatus* (6 replicates) feeding on green vs. red parts of *Cladonia rubrotincta*.

Generalized Mixed Model

Model Info

Info	Value	Comment
Model Type	Poisson	Model for count data
Call	glm	pellets ~ 1 + lichen + mite + day + lichen:mite + (0 + day | box)
Link function	log	Coefficients are in the log(y) scale
Distribution	Poisson	Model for count data
LogLikel.	-2827.769	Unconditional Log-Likelihood
-2*LogLikel.	5655.538	Unconditional absolute deviance
Deviance	4830.309	Conditional relative deviance
R-squared	0.901	Marginal
R-squared	0.971	Conditional
AIC	5667.540	Less is better
BIC	5687.447	Less is better
Residual DF	198.000	
Chi-squared/DF	22.092	Overdispersion indicator
Converged	yes	
Optimizer	bobyqa	

Model Results

Fixed Effect Omnibus tests

	X^2	df	p
**lichen**	1811.2	1.00	<.001
**mite**	143.8	1.00	<.001
**day**	62.8	1.00	<.001
**lichen ✻ mite**	336.2	1.00	<.001

Fixed Effects Parameter Estimates

Names	Effect	Estimate	SE	exp(B)	95% Exp(B) Confidence Interval		z	p
					Lower	Upper		
(Intercept)	(Intercept)	3.089	0.0182	21.953	21.182	22.751	169.50	<.001
lichen	green – **red***	-1.325	0.0311	0.266	0.250	0.283	-42.56	<.001
mite	marginatus - areolatus	0.436	0.0364	1.547	1.440	1.661	11.99	<.001
day	day	0.183	0.0231	1.201	1.148	1.256	7.93	<.001
lichen ✻ mite	Without - Spots ✻ marginatus - areolatus	1.142	0.0623	3.132	2.772	3.539	18.34	<.001

* Preferred lichen printed in bold.

Random Components

Groups	Name	SD	Variance	ICC
**replicate**	**day**	0.0723	0.00522	
**Residuals**		1.0000	1.00000	.

Note. Number of Obs: 204 , groups: 10 replicates (4 areolatus +6 marginatus)

Post Hoc Tests

Post Hoc Comparisons - lichen ✻ mite

Comparison								
**lichen**	**mite**		**lichen**	**mite**	**exp(B)**	**SE**	**z**	**$p_{bonferroni}$**
red	areolatus	-	red	marginatus	1.144	0.03727	4.14	<.001
red	areolatus	-	green	areolatus	6.658	0.36238	34.83	<.001
red	areolatus	-	green	marginatus	2.432	0.09070	23.84	<.001
red	marginatus	-	green	marginatus	2.126	0.06429	24.93	<.001
green	areolatus	-	red	marginatus	0.172	0.00971	-31.16	<.001
green	areolatus	-	red	marginatus	0.365	0.02169	-16.96	<.001

Post Hoc Comparisons - mite

Comparison						
mite		mite	exp(B)	SE	z	$p_{bonferroni}$
areolatus	-	marginatus	0.647	0.0235	-12.0	<.001

References:

The jamovi project (2024). *jamovi*. (Version 2.6) [Computer Software]. Retrieved from https://www.jamovi.org.

R Core Team (2024). *R: A Language and environment for statistical computing*. (Version 4.4) [Computer software]. Retrieved from https://cran.r-project.org. (R packages retrieved from CRAN snapshot 2024-08-07).

Gallucci, M. (2019). *GAMLj: General analyses for linear models*. [jamovi module]. Retrieved from https://gamlj.github.io/.
